# Blood Flow Velocity Analysis in Cerebral Perforating Arteries on 7T 2D Phase Contrast MRI with an Open-Source Software Tool (SELMA)

**DOI:** 10.1007/s12021-024-09703-4

**Published:** 2025-01-22

**Authors:** S. D. T. Pham, C. Chatziantoniou, J. T. van Vliet, R. J. van Tuijl, M. Bulk, M. Costagli, L. de Rochefort, O. Kraff, M. E. Ladd, K. Pine, I. Ronen, J. C. W. Siero, M. Tosetti, A. Villringer, G. J. Biessels, J. J. M. Zwanenburg

**Affiliations:** 1 Translational Neuroimaging Group, Center for Image Sciences, University Medical Center Utrecht, Utrecht University, Utrecht, The Netherlands; 2 Division Imaging & Oncology, Image Sciences Institute, UMC Utrecht, Utrecht, The Netherlands; 3 Department of Radiology, University Medical Center Leiden, Leiden, The Netherlands; 4 Department of Neuroscience, Genetics, Maternal and Child Health, University of Genoa, RehabilitationGenoa, Ophthalmology Italy; 5 IRCCS Ospedale Policlinico San Martino, Genoa, Italy; 6 Aix Marseille University, CNRS, CRMBM, Marseille, France; 7 Erwin L. Hahn Institute for Magnetic Resonance Imaging, University of Duisburg-Essen, Essen, Germany; 8 Medical Physics in Radiology, German Cancer Research Center (DKFZ), Heidelberg, Germany; 9 Department of Neurophysics, Max Planck Institute for Human Cognitive and Brain Sciences, Leipzig, Germany; 10 Brighton and Sussex Medical School, University of Sussex, Falmer, UK; 11 Spinoza Centre for Neuroimaging, Amsterdam, Netherlands; 12 Laboratory of Medical Physics and Magnetic Resonance, IRCCS Stella Maris, Pisa, Italy; 13 Department of Neurology, Max Planck Institute for Human Cognitive and Brain Sciences, Leipzig, Germany; 14 Department of Neurology and Neurosurgery, UMC Utrecht Brain Center, University Medical Center Utrecht, Utrecht University, Utrecht, The Netherlands

**Keywords:** Pulsatility index, Blood flow velocity, Perforating arteries, Analysis tool, 2D PC-MRI

## Abstract

Blood flow velocity in the cerebral perforating arteries can be quantified in a two-dimensional plane with phase contrast magnetic imaging (2D PC-MRI). The velocity pulsatility index (PI) can inform on the stiffness of these perforating arteries, which is related to several cerebrovascular diseases. Currently, there is no open-source analysis tool for 2D PC-MRI data from these small vessels, impeding the usage of these measurements. In this study we present the Small vessEL MArker (SELMA) analysis software as a novel, user-friendly, open-source tool for velocity analysis in cerebral perforating arteries. The implementation of the analysis algorithm in SELMA was validated against previously published data with a Bland–Altman analysis. The inter-rater reliability of SELMA was assessed on PC-MRI data of sixty participants from three MRI vendors between eight different sites. The mean velocity (v_mean_) and velocity PI of SELMA was very similar to the original results (v_mean_: mean difference ± standard deviation: 0.1 ± 0.8 cm/s; velocity PI: mean difference ± standard deviation: 0.01 ± 0.1) despite the slightly higher number of detected vessels in SELMA (N_detected_: mean difference ± standard deviation: 4 ± 9 vessels), which can be explained by the vessel selection paradigm of SELMA. The Dice Similarity Coefficient of drawn regions of interest between two operators using SELMA was 0.91 (range 0.69–0.95) and the overall intra-class coefficient for N_detected_, v_mean_, and velocity PI were 0.92, 0.84, and 0.85, respectively. The differences in the outcome measures was higher between sites than vendors, indicating the challenges in harmonizing the 2D PC-MRI sequence even across sites with the same vendor. We show that SELMA is a consistent and user-friendly analysis tool for small cerebral vessels.

**Supplementary Information:**

The online version contains supplementary material available at 10.1007/s12021-024-09703-4.

## Introduction

Cerebral blood flow in large intracranial arteries can be quantified using phase contrast magnetic resonance imaging (PC-MRI). This technique estimates the blood flow velocity from the MR signal's phase accrual of moving spins as the velocity of the moving spins (i.e. blood) is proportional to this phase accrual. Recently, with the advantages of the increased sensitivity on high-field 7T MRI, granting higher spatial resolutions with adequate SNR, blood flow velocity of the brain's perforating arteries through a two-dimensional plane can be measured using 2D PC-MRI (Bouvy et al., 2016; Zwanenburg & Osch, 2017).

Blood flow velocity measurements can inform on the condition of these arteries, notably also in disease states (Björnfot et al., 2024; Rivera-Rivera et al., 2017; Vikner et al., 2020; Zarrinkoob et al., 2016). In particular, the blood flow velocity's pulsatility index (PI) is an important indicator of arterial stiffness (Mitchell et al., 2011; O'Rourke & Hashimoto, 2007; Webb et al., 2012). Increased stiffness in the large intracranial arteries has been associated with damage to the brain parenchyma, such as microbleeds (Sloten et al., 2015; Zhai et al., 2018), lacunar infarcts (Chuang et al., 2016; Sloten et al., 2015), and white matter hyperintensities (Aribisala et al., 2014; Poels et al., 2012; Sloten et al., 2015), while also being linked to cognitive impairment (Mitchell et al., 2011; Singer et al., 2014) and cognitive decline (Singer et al., 2014; Zeki Al Hazzouri et al., 2013). In the smaller arteries, information from these blood flow velocity measurements could be especially relevant in cerebral small vessel diseases (cSVDs), an important cause of stroke and dementia (Wardlaw et al., 2019). Recently, using 7T MRI, an increased velocity PI in the perforating arteries was found in patients with cSVDs and a lower blood flow velocity was linked to white matter damage increase/progression (Brink et al., 2023; Brink et al., 2024). Age was also found to be a determinant of increased velocity PI in the perforating arteries of the basal ganglia in both cSVDs and general population cohorts (Perosa et al., 2022; Schnerr et al., 2017; Vikner et al., 2020).

For the larger intracranial arteries, several publicly available image processing tools exist to analyze PC-MRI data (Hespen et al., 2022; Köhler et al., 2017, 2019; Roberts et al., 2023). For the smaller arteries, however, there are no processing tools available yet. Reported analyses of PC-MRI data of the smaller arteries were performed with a collection of in-house developed code (Arts et al., 2021a; Bouvy et al., 2016; Kerkhof et al., 2023a), which has several limitations. First, such code consists of scripts in which the parameters of the algorithms are hard-coded and not accessible as user-defined settings. Second, these scripts, including parameter settings, often evolve with time without explicit version tracking. Also, as part of the developments of the analysis methods, several variants of output parameters were computed in parallel, such as the PI from the mean normalized velocity traces from all arteries and the mean over the PIs of the individual velocity traces. This situation can potentially create ambiguities and unintentionally result in inconsistent application of output parameters in studies. Additionally, it impedes the reproducibility of previous analyses, even more so across centers. Finally, the lack of version-controlled software with a user-friendly user interface hampers the dissemination of the methods to other researchers, which limits the usage of blood flow velocity and PI measurements in the perforating arteries of the basal ganglia and semioval center on a larger scale. To support consistent, repeatable analysis and to promote the usage and possibly further development of the PC-MRI measurements in small arteries, an analysis tool should be established that is accessible (open-source), user-friendly (with a graphical user interface (GUI)), and facilitate repeatable analysis (logging of version and settings with results) of small artery PC-MRI data.

This work presents the Small vessEL MArker (SELMA) analysis software as a novel, open-source tool for cerebral small artery flow velocity analysis compatible with data from multiple MRI vendors. SELMA incorporates converged and updated analysis algorithms used in our previous publications (Arts et al., 2021a; Bouvy et al., 2016; Geurts et al., 2018). To validate the implementation of the algorithm in SELMA, we re-analyzed previously published data (Arts et al., 2021b) and compared the results of SELMA with the original results obtained with a previously published iteration of the algorithm. Additionally, we assessed the inter-rater reliability of SELMA on data from three different 7T MRI vendors by two trained operators. A secondary aim was to assess the performance of measuring perforating artery velocity pulsatility at MRI scanners from different sites and vendors by comparing measurements between sites and testing for age and sex effects.

## Methods

### Algorithm

**Fig. 1:**
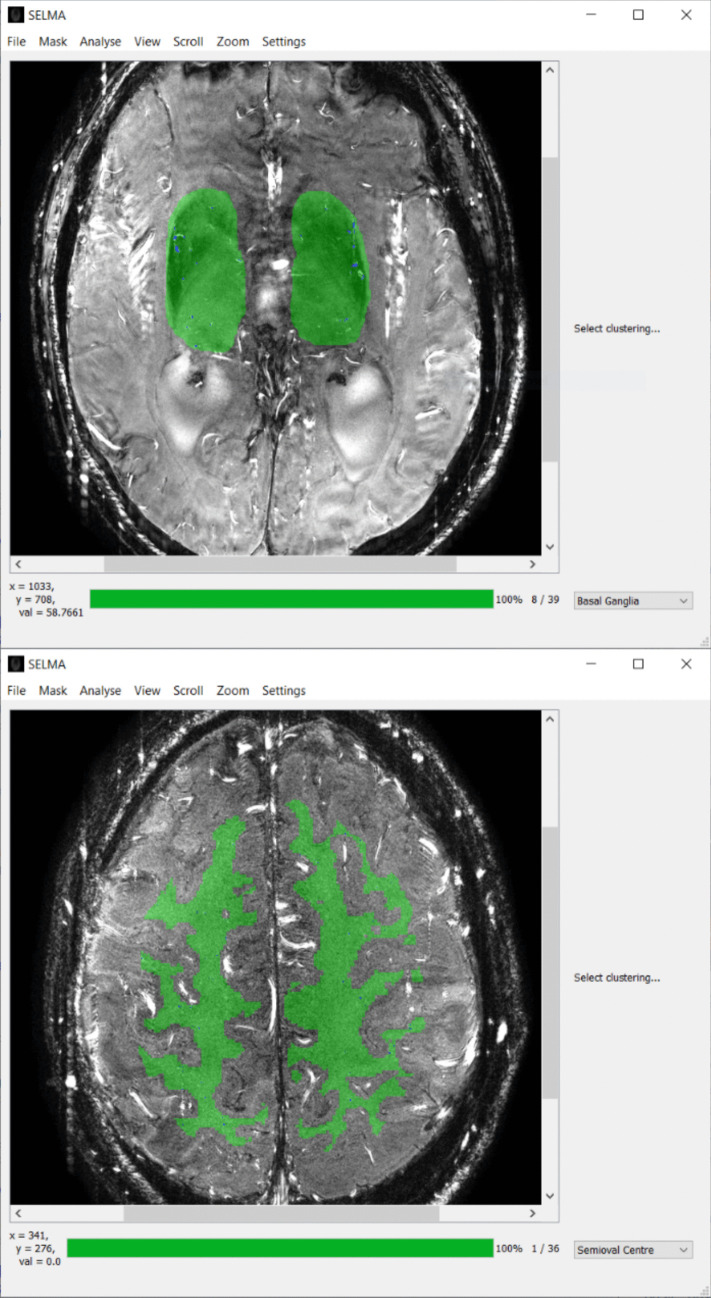
The graphical user interface of SELMA after analysis has been completed in the basal ganglia (top) or center semioval (bottom). Slice selection can be performed in the bottom right. Note that it is important to verify that the correct slice is selected as that will change certain algorithm parameters. Analysis is initiated by either manually drawing or loading in a pre-defined region of interest (ROI) such as a white matter mask. ROIs can be directly drawn in SELMA and saved for further analysis. After completion, SELMA will highlight detected vessels in blue and automatically produce an output file containing the outcome measurements for every vessel and averaged over all vessels. SELMA settings can be accessed in the top bar of the GUI

The original algorithm used to analyze small artery PC-MRI data as described in previous work (Arts et al., 2021a; Bouvy et al., 2016; Geurts et al., 2018) was developed in MATLAB (version R2015b, the MathWorks, Natick, MA). These MATLAB scripts have been rewritten in Python 3.7 to develop SELMA for analysis of the cerebral perforating arteries at the level of the basal ganglia or the white matter at the semioval center (Fig. 1). SELMA was developed to be compatible with all 2D PC-MRI DICOM data, regardless of MRI vendor or field strength. The implementation of the algorithm in SELMA follows that of the original codes (Arts et al., 2021a; Bouvy et al., 2016; Geurts et al., 2018), but includes several updates and improvements, which will be detailed below.

Analysis in SELMA can be started by drawing a region of interest (ROI) on the 2D PC-MRI images using the software interface or loading such mask from the disk. The first step of the analysis is the estimation of the noise level in the phase and magnitude frames, which is computed on a voxel-wise basis using the standard deviation of the complex signal (constructed from the phase and magnitude data) over the cardiac cycle. The root mean square of the standard deviations of the imaginary and complex parts of the constructed complex signal are computed next. A median filter with a user-defined kernel size (default 10 mm) is then applied to the root mean square maps. The temporal signal-to-noise ratio of the magnitude frames (SNR_mag_) is computed by dividing the magnitude frames with the median filtered root mean square maps. The phase maps are scaled to velocity maps with the pre-defined velocity encoding (v_enc_) of the scan protocol parameters available from the DICOM header. The velocity frames are averaged over the cardiac cycle and median-filtered with a 10 mm kernel. The median filtered velocity map is subtracted from the raw velocity maps at each point in the cardiac cycle to remove the background phase of static tissue and to center the velocity around zero. Next, using SNR_mag_, the standard deviation of the corrected velocity maps (σ_v_) can be computed using the following formula (Conturo & Smith, 1990):

$${\sigma }_{v}=\frac{{v}_{enc}}{\pi }\frac{1}{{SNR}_{mag}}$$

The corrected velocity maps are divided by σ_v_ to obtain the temporal signal-to-noise ratio of the velocity frames (SNR_v_). Based on the SNR_mag_ and SNR_v_, voxels in the 2D PC-MRI image can be clustered into several profiles (Table 1). For the detection of vessels in the basal ganglia, clusters of voxels with an SNR_mag_ and SNR_v_ above a positive noise threshold (T_n_) are detected as potential arteries. Clusters of voxels in the semioval center are identified as arteries only if SNR_v_ is lower than the negative T_n_, regardless of SNR_mag_. The sign for SNR_v_ in the semioval center is changed due to the opposite directionality of the blood flow of the perforating arteries at the semioval center compared to the basal ganglia. T_n_ can be changed by the user by changing the significance level in the settings (default 0.05, in which case T_n_ = 1.96). A new addition in SELMA is the scaling of T_n_ based on the number of acquired heart phases and the temporal resolution of the data. Estimation of the SNR in the magnitude and velocity frames are dependent on the heart rate, which affects the number of acquired heart phases, and on the number of k-space lines acquired per heartbeat and TR of the acquisition, which affects the temporal resolution.

**Table 1:** Standard clustering profiles for basal ganglia and semioval center 2D PC-MRI scans in SELMA

	SNR_vel_ <—T_n_	- T_n_ < SNR_vel_ < T_n_	SNR_vel_ > T_n_
Basal ganglia			
SNR_mag_ > T_n_	-	-	+
SNR_mag_ < T_n_	-	-	-
Semioval center			
SNR_mag_ > T_n_	+	-	-
SNR_mag_ < T_n_	+	-	-

Clusters of voxels are only detected as vessel in the basal ganglia if both SNR_mag_ and SNR_vel_ exceed T_n_

In the centrum semioval, voxels with a SNR_vel_ lower than –T_n_ (note the sign change because of the different direction of the flow) are detected as vessel, regardless of SNR_mag_. SNR_mag_ = magnitude signal-to-noise ratio; SNR_vel_ = velocity signal-to-noise ratio; T_n_ = noise threshold.

After identification of the arteries that exceed T_n_, operators of SELMA can select the options to automatically filter out arteries whose orientation is far from being perpendicular to the scanning plane and/or to deduplicate arteries that are too close to each other. The blood flow velocity in arteries that are not perpendicular to the scanning plane will be underestimated (Bouvy et al., 2016). SELMA considers the artery's roundness to determine whether the artery can be considered as perpendicular to the scanning plane, at least in first approximation, in which case it should be included in the analysis. An ellipse is fitted to the artery's circumference, and the axes ratio of the ellipse is computed, which discards the artery from further analysis if the axes ratio exceeds a user-set threshold (default 2). Artery detections that are too close to each other might be multiple detections on a single artery oriented parallel to the scanning plane or might be caused by ghosting artefacts. In such cases, SELMA discards all arteries except the one with the highest velocity if detected within a user-set distance (default 1.2 mm) from each other. These options can be applied both to basal ganglia and semioval center focussed PC-MRI scans. Additional options can be selected to erode the outer region of the ROI and/or filter out ghosting artefacts from the scans. Arteries in the outer edges of the ROI in the semioval center are more prone to motion artifacts. Users can specify how many voxels from the edges of the ROI can be eroded (default 80). Ghosting of larger arteries in the phase encoding direction can lead to erroneous artery detections. SELMA incorporates the automatic ghosting censoring method as previously described (Arts et al., 2021a). First, SELMA identifies the large blood arteries by applying a relative intensity threshold on the magnitude images. Only clusters of voxels in the top magnitude percentile defined by the user (default 0.3%) and larger than a minimum size are included as potential large arteries. Depending on the size of the bright voxel cluster, an exclusion zone will be drawn around the cluster. Undesired detections in these zones will be discarded from further analysis. Ghosting artefacts are a larger issue in the semioval center when automatically segmented ROIs are used compared to the basal ganglia where they can be avoided when manually delineating a ROI. In the options, users can define custom values for the thresholds, voxel sizes of the arteries or length and width of the exclusion zones.

Another addition to SELMA is the ability for operators to censor artery detections in the basal ganglia manually. Selecting this option will override the in-plane artery censoring and deduplication steps. SELMA will visualize all detected arteries and guide the user systematically through every artery prompting the user if it should be discarded for analysis. The user can see the proximity of the artery to others in the user interface, and the axes ratio of the artery is provided to aid the user in this process. The user can opt to keep or discard the highlighted artery, and is then guided to the next one. After all detected arteries have been evaluated, SELMA prompts the user to either save their results, or to restart the manual vessel censoring process again from the beginning. All parts of the manual vessel censoring functionality can be directly performed in the GUI.

After final artery selection, the number of perforating arteries (N_detected_), their mean blood flow velocity (v_mean_) and the velocity PI are assessed. The PI is generally defined as $\frac{{v}_{max}-{v}_{min}}{{v}_{mean}}$, where v_max_, v_min_, and v_mean_, are the maximum, minimum, and mean of the velocity trace over the cardiac cycle in cm/s. We calculated the PI from the averaged velocity trace of all final included arteries, while the individual velocity traces were normalized before averaging (hence, v_mean_ in the PI formula was 1.0 by definition). During the development of SELMA, an approach using the median of the normalized velocity trace was also considered, however during internal testing, we found that the blood flow velocity distribution over all arteries for each time point in the cardiac cycle followed a normal distribution and that using a median estimator had a higher uncertainty than the mean estimator, which led to inflated values of the PI (Brink et al., 2024). Thus, we opted to use the mean of the normalized velocity trace to compute the PI. During internal testing, we observed similar group differences in blood flow velocity and velocity PI using the mean versus median normalized velocity trace.

### User Interface

Analysis of small vessel 2D PC-MRI data has been made more user friendly with the addition of a GUI in SELMA (Fig. 1). 2D PC-MRI scans of either the basal ganglia or the semioval center in DICOM format can be loaded into SELMA via the menu in the top bar. ROIs that were either manually drawn in SELMA or segmented with external software, such as white matter masks, can also be loaded from the disk with this menu. Analysis can be started with the 'Analyse' option in the menu for single scans or, if masks are already provided, entire folders containing scans. The batch analysis option in SELMA allows for automatic analysis of multiple scans without any user input, drastically speeding up the analysis process. Two default voxel clustering settings have been defined, tailored to acquisitions at the basal ganglia and semioval center, respectively. Before the analysis, the actual slice location must be selected in the bottom right-hand corner to ensure that the corresponding predefined voxel clustering settings are applied in SELMA. Custom clustering options can also be selected in this menu for analysis of data that do not fall into the standard profiles for basal ganglia or semioval center scans. The remaining options in the top menu allow for changing the contrast or zoom of the image, which can also be directly manipulated in the user interface using the mouse. In the settings menu, the operator can change several options and thresholds as described in the 'Algorithm' section.

SELMA was developed specifically with the goal of an easy to maintain code base. To this end, the GUI and the processing parts of the code are intentionally separated in different classes that can only talk to each other using the Signal-and-Slot paradigm. This should enable future developers to easily understand the code, and make small changes to either the processing algorithms or the GUI without accidentally introducing unwanted behavior elsewhere in the program.

### Test Data

**Fig. 2:**
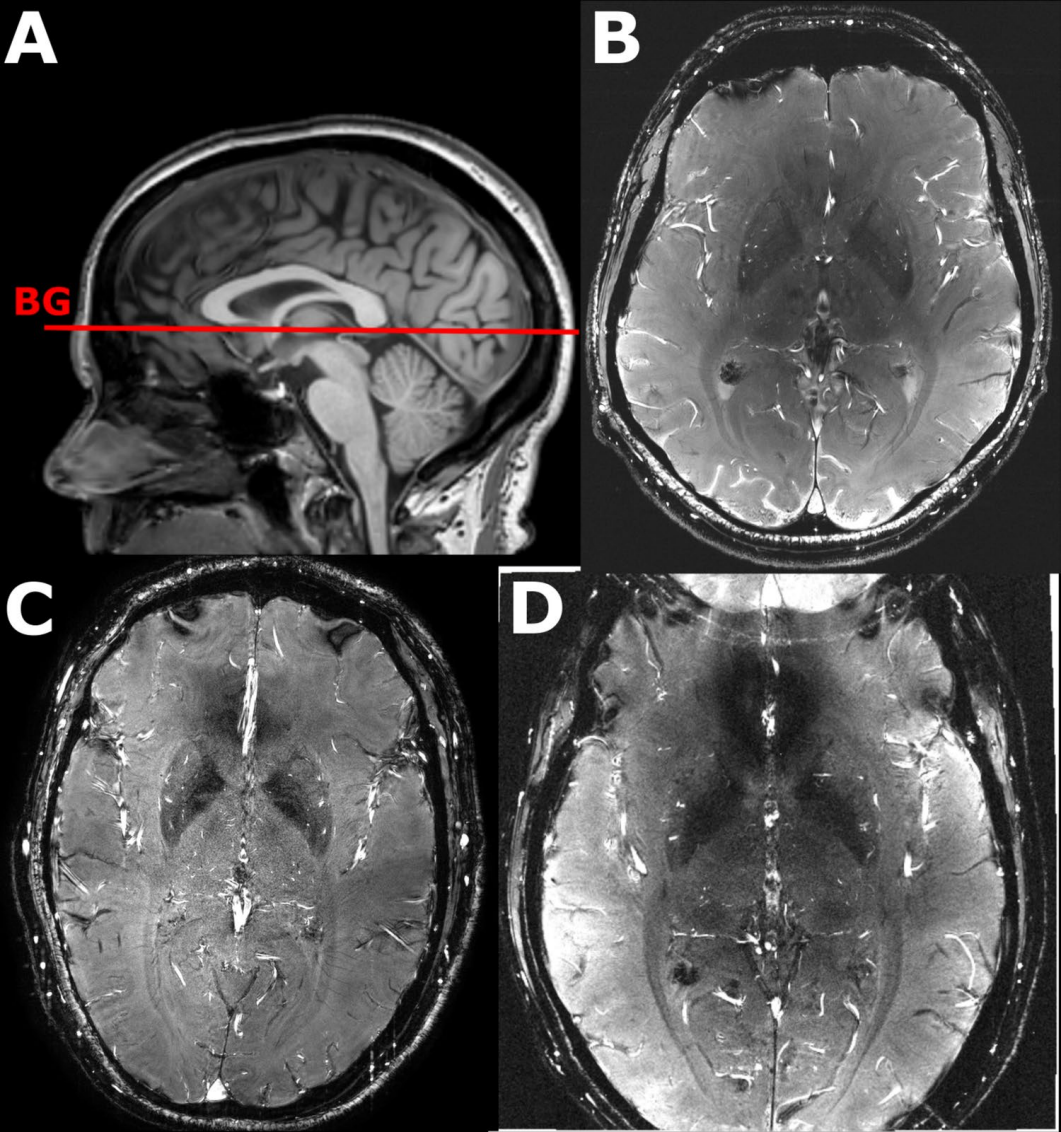
**A**) The two dimensional phase contrast (2D PC) acquisition was planned on a 3D anatomic image (T_1_) at the level of the basal ganglia. Images **B**, **C** and **D** show representative magnitude scans of the basal ganglia in three different subjects for vendor 1, 2, and 3 respectively. To achieve the same spatial resolution across vendors, the image in panel D was acquired with a smaller field-of-view due to setting limitations in the acquisition matrix size. The unavoidable aliasing in the anterior–posterior direction does not affect the analysis in the region of interest

The implementation of the analysis algorithm in SELMA was validated with 7T MRI (Achieva 7T, Philips Medical Systems, Best, The Netherlands) data from previous work (Arts et al., 2021b). These 2D PC-MRI data were acquired with a 32-channel receive head coil (Nova Medical, Wilmington, NC, United States) in 14 patients with coarctation of the aorta and 15 control subjects with no history of cardiovascular disease, neurological disease, or intellectual disability. The 2D PC sequence was planned on a 3D T1-weighted image at the level of the basal ganglia (Fig. 2) and was retrospectively gated using a peripheral pulse oximeter for triggering. The following scan parameters were used: 250 × 250 mm^2^ field of view; acquired spatial resolution 0.3 × 0.3 × 2.0 mm^3^, reconstructed spatial resolution (through zero-filling in k-space) 0.2 × 0.2 × 2.0 mm^3^; TR/TE = 28/14.7–15.1 ms; flip angle = 50°; TFE factor = 2 (i.e. number of k-lines acquired per cardiac frame, per heart beat); velocity encoding = 20 cm/s; acquired temporal resolution = 112 ms; 13–15 reconstructed heart phases, depending on the heart rate; SENSE factor = 1.5; scan duration was about 5 min for a heart rate of 60 beats/min.

Inter-rater reliability was assessed with data from 8 different scanning sites comprising three different MRI vendors: Philips (Achieva 7T, Philips Healthcare, Best, The Netherlands), Siemens (MAGNETOM 7T, Siemens Healthineers, Erlangen, Germany) and General Electric (GE) (Discovery MR950, GE Healthcare, Chicago, Illinois, USA). All MRI data were acquired with a 32-channel receive head coil (Nova Medical, Wilmington, NC, United States), except for one site where a 24-channel receive head coil (Nova Medical, Wilmington, NC, United States) was used. All scanning sites participated in the European Ultrahigh-Field Imaging Network for Neurodegenerative Diseases (EUFIND) (Düzel et al., 2019). EUFIND comprises researchers from 22 7T MRI sites in Europe with the common aim of identifying opportunities and challenges of 7T MRI for clinical and research applications in neurodegeneration. Each site included healthy elderly subjects aged between 50 and 70 years with an equal gender distribution and attempted to include an equal number of volunteers between 50 and 60 years and between 60 and 70 years to allow for secondary analysis of age and gender effects on the outcome parameters. All institutions performed the scanning under the approval of the institution's local ethical review board, and all subjects provided written informed consent.

The 2D PC-MRI sequence was originally developed for the Philips 7T MRI and the protocol was harmonized across all sites with the parameters in Table 2. Harmonization was limited by the vendor-specific implementation of the 2D PC sequence and parameter ranges. The acquisition slice was planned in the basal ganglia, targeting the perforating lenticulostriate arteries that branch from the circle of Willis. Representative basal ganglia scans per vendor are shown in Fig. 2.

**Table 2:** Desired scan parameters for the two dimensional phase contrast sequence for all three MRI vendors

FOV RL x AP (mm)	250 × 250
Foldover direction	AP
Acquired matrix	832 × 833
Reconstructed voxel size (mm)	0.2 × 0.2
Parallel imaging	none
Slice thickness (mm)	2.0
TFE factor (nr. phase enc. lines per cardiac time point)	2
TR/TE (ms)	27/16
Flip angle	60°
Cardiac synchronization	Retrospective gating
Reconstructed nr. of heart phases	14
Acquired temporal resolution	107 ms
Venc (cm/s)	20
Scan duration	4 min with heart rate of 60 bpm

### Data Analysis

The differences in the outcome measures between SELMA and the previously published results on the validation data were quantified on a group level with a Bland–Altman analysis. Inter-rater reliability of the ROIs drawn in SELMA was assessed using the Dice similarity coefficient (DSC) between ROIs drawn by two trained operators. Inter-rater reliability of the outcome measures using manual artery selection was assessed by calculating the Intra-class Correlation Coefficient (ICC) between two operators. An ICC above 0.75 was considered to be an excellent correlation between operators (Cicchetti, 1994). The coefficient of variation (CV) of all outcome measures and the SNR_v_ of the included arteries averaged over all subjects within each site were used to assess inter-site differences. The CV for all sites within a single vendor was computed and subsequently averaged over all vendors to assess the inter-vendor differences. The relation of age and sex with the outcome measures was assessed using a linear mixed model corrected with site and vendor as random effects. All statistical analyses were performed in MATLAB (version R2021a, the MathWorks, Natick, MA) except for the linear mixed modelling, which was performed in R (version 4.2.1, R Foundation for Statistical Computing, Vienna, Austria) with the 'lme4' package.

## Results

### Validation of the Implementation

Supplementary Table 1 shows the results of the original measurements compared to SELMA on previously published data of 29 participants (Köhler et al., 2019). In the same data, SELMA had a slightly higher N_detected_ compared to the previous version of the algorithm. The v_mean_ and velocity PI were similar between both approaches at a group level. Bland–Altman plots for the comparison between the original measurements and SELMA are shown in Supplementary Fig. 1.

### Multi-Vendor Comparison

Data of 60 participants (mean age ± standard deviation: 59 ± 6 years) were included (eight sites, three MRI vendors) for the multi-vendor analysis. Due to vendor-specific implementation of the 2D PC sequence, scan parameters slightly varied between subject, site and vendor. Table 3 provides an overview of the relevant parameters. The N_detected_, v_mean,_ PI, and SNR_v_ are stratified per vendor in Table 4 and per site in Table 5. The CV for inter-site differences were larger for all measurements than the inter-vendor differences (Table 6).

**Table 3:** Scan parameters (mean ± SD) for the two-dimensional phase-contrast sequence for all three MRI vendors

Scan parameter	Vendor 1	Vendor 2	Vendor 3
Head coil	32-channel receiver Circular polarized transmit coil	32-channel receiver Circular polarized transmit coil	24-channel / 32-channel receiver
FOV, mm	242 ± 4 × 242 ± 4	250 × 250	154 ± 0.4 × 154 ± 0.4
Reconstructed voxel size, mm	0.23 × 0.23	0.2 × 0.2	0.3 × 0.3
Flip angle, °	60	60	54–60
TR/TE, ms	32.4 ± 1.8 / 11.3 ± 0.5	26.3 ± 0.4 / 15.9 ± 0.4	28.1 ± 1.1 / 17.2 ± 0.4
Bandwidth, Hz/pixel	78	59	60.8 ± 4.2
Echo Train Length	2	2—3	2
Acquired temporal resolution, ms	129.6 ± 7.2	128.7 ± 27.3	112.1 ± 4.3
Time points	12 ± 1	14 ± 1	14
Scan time, seconds	384 ± 72	242 ± 73	246 ± 39
Average heartrate/min	68 ± 16	60 ± 11	66 ± 10

**Table 4:** Outcome measures and inter-rater results per vendor

	Vendor 1 (n = 10)	Vendor 2 (n = 20)	Vendor 3 (n = 30)	All (n = 60)
Outcome measures				
N_detected_ (n)	4 ± 3	18 ± 5	7 ± 5	10 ± 7
v_mean_ (cm/s)	3.6 ± 1.3	3.8 ± 0.7	5.7 ± 1.4	4.7 ± 1.5
PI	0.87 ± 0.27	0.36 ± 0.11	0.46 ± 0.24	0.49 ± 0.27
SNR_v_	5.9 ± 2.5	6.7 ± 2.2	5.3 ± 1.5	5.9 ± 2.0
Inter-rater analysis				
DSC	0.88 [0.81—0.93]	0.93 [0.90—0.95]	0.90 [0.69—0.95]	0.91 [0.69—0.95]
ICC N_detected_	0.75 [0.22—0.94]	0.72 [0.43—0.88]	0.88 [0.76—0.94]	0.92 [0.87—0.95]
ICC v_mean_	0.80 [0.21—0.96]	0.75 [0.47—0.89]	0.76 [0.54—0.88]	0.84 [0.74—0.90]
ICC PI	0.32 [−0.50—0.84]	0.79 [0.54—0.91]	0.93 [0.86—0.97]	0.85 [0.76—0.91]

Values are given in mean ± SD for the outcome measures and mean with 95% confidence interval for the inter-rater analysis. N_detected_ = amount of detected arteries; v_mean_ = mean blood flow velocity of the perforating arteries given in cm/s; PI = pulsatility index; ICC = intra-class coefficient; DSC = Dice similarity coefficient

**Table 5:** Outcome measures per site

Measurement	Site 1 (n = 10)	Site 2 (n = 2)	Site 3 (n = 9)	Site 4 (n = 9)	Site 5 (n = 5)	Site 6 (n = 6)	Site 7 (n = 10)	Site 8 (n = 9)
N_detected_ (n)	4 ± 3	17 ± 4	16 ± 5	21 ± 3	3 ± 2	4 ± 4	11 ± 3	7 ± 4
v_mean (_cm/s)	3.6 ± 1.3	3.0 ± 0.3	4.0 ± 0.7	3.8 ± 0.5	6.6 ± 1.6	5.4 ± 1.2	4.9 ± 0.8	6.1 ± 1.6
PI	0.87 ± 0.27	0.43 ± 0.27	0.36 ± 0.11	0.34 ± 0.07	0.59 ± 0.56	0.46 ± 0.05	0.42 ± 0.14	0.45 ± 0.10
SNR_v_	5.9 ± 2.5	4.5 ± 2.0	7.6 ± 2.7	6.3 ± 1.2	5.1 ± 1.4	4.1 ± 0.8	6.2 ± 1.7	5.1 ± 1.2

Values are given as mean ± SD. N_detected_ = amount of detected arteries; v_mean_ = mean blood flow velocity of the perforating arteries given in cm/s and PI = pulsatility index

**Table 6:** Coefficient of variation for inter-site and inter-vendor measurements

Measurement	Inter-site	Inter-vendor
N_detected_ (n)	62	31
v_mean_ (cm/s)	21	12
PI	39	12

N_detected_ = amount of detected arteries; v_mean_ = mean blood flow velocity of the perforating arteries given in cm/s; PI = pulsatility index; CV = coefficient of variation. Coefficients of variation are given as percentages

### Inter-Rater Analysis

The mean DSC for the manually drawn ROIs by the two operators was 0.91 (range 0.69–0.95) across all 60 participants (Table 4). All ICCs for the measurements exceeded 0.75 except for the velocity PI of vendor 1 and N_detected_ of vendor 2. The ICC of N_detected_, v_mean_, and velocity PI for the entire dataset was 0.92, 0.84, and 0.85, respectively (Table 4).

### Age and Gender Analyses

**Fig. 3:**
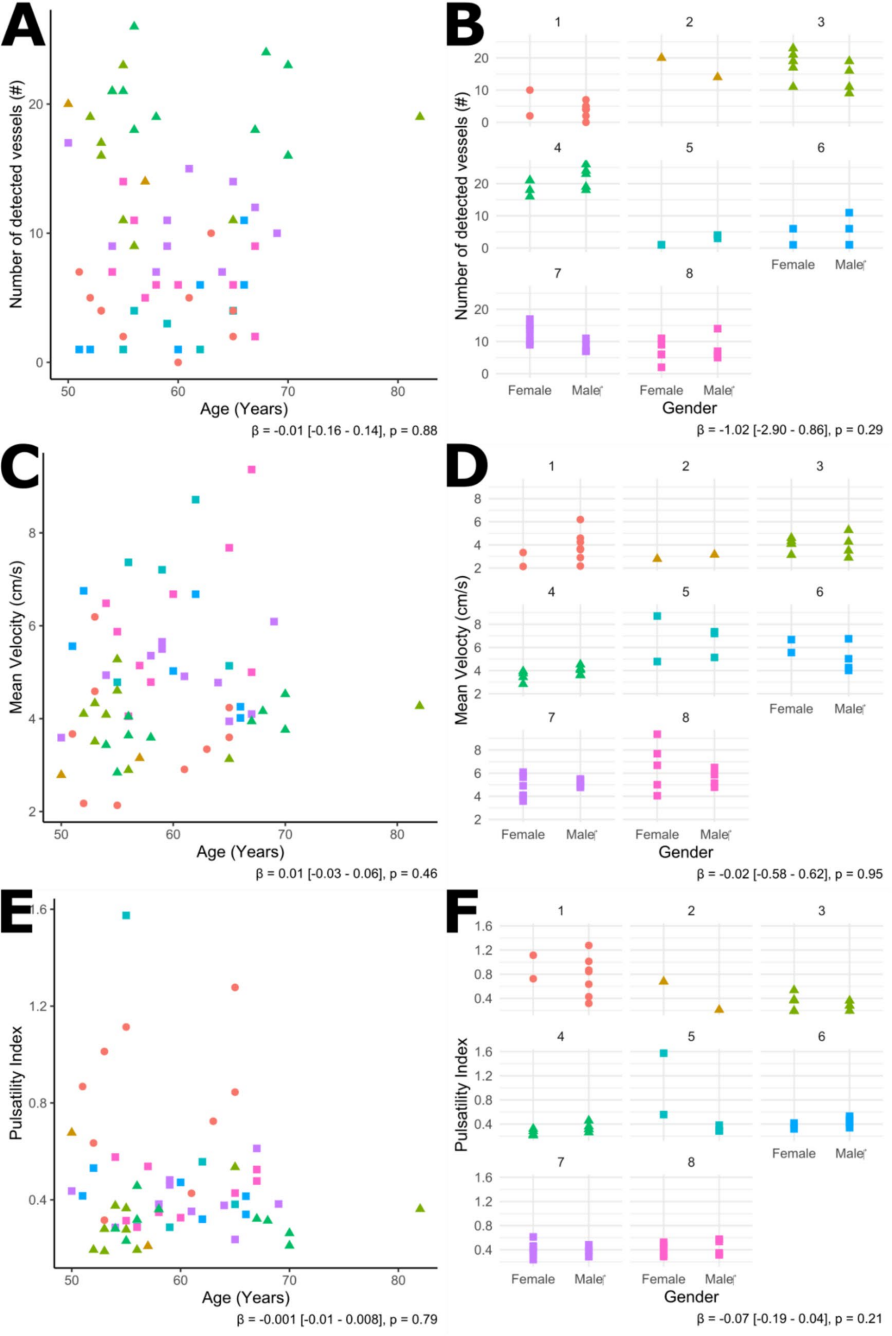
The scatterplots on the left show the relation between age and N_detected_ (**A**), v_mean_ (C), and PI (E) respectively. The plots on the right show the differences between females and males for N_detected_ (B), v_mean_ (D), and PI (F) respectively (8 plots for the 8 sites). The different shapes and colors in the plots represent the different vendors and sites respectively. The regression coefficient with respective 95% confidence interval and p-value is shown under each plot for each linear mixed model with age or gender and the outcome measures, including site and vendor as co-variates. Regression coefficients for age are given per year and for gender with female as reference (i.e. female = 0, male = 1). N_detected_ = number of detected arteries; v_mean_ = mean blood flow velocity of the perforating arteries given in cm/s; PI = pulsatility index

Age and gender were not significantly associated with N_detected_, v_mean_, and velocity PI over the entire population in multivariate models adjusted for site and vendor (Fig. 3).

## Discussion

In this study, we developed SELMA, an open-source small cerebral artery analysis tool for 2D PC-MRI data. We have shown that the results using SELMA match well with the results of the original version of our algorithm using published data (Arts et al., 2021b). Reproducible multi-vendor analysis of 2D PC-MRI data was also possible with SELMA.

With SELMA, we obtained a higher N_detected_ than in the previous analysis, which can be explained by the changes that were made in the deduplication steps. Previously, all perforating arteries within a 1.2 mm distance from each other were discarded in the analysis, which led to more false negatives during detection. In SELMA, only the artery with the highest v_mean_ is kept, and all other detections within a 1.2 mm distance are discarded. The increase in N_detected_ had no effect on the average v_mean_ and velocity PI, as these were similar to the original results over the entire group. This supports the original assumption that averaging over all available small arteries gives a representative number for the detected population of arteries (Bouvy et al., 2016). The Limits of Agreement (LoA) for the velocity PI found in the Bland–Altman analysis was similar to the LoA on a group-level for the original measurements on 7T (LoA original versus SELMA: 0.20 vs 0.16) (Köhler et al., 2019), suggesting that the variability in velocity PI can mostly be explained by thermal or physiological noise during the measurement rather than a bias introduced by different analysis methods.

On multi-vendor data, analysis with SELMA showed that the CV in the outcome measures was higher between sites than between vendors. The CV for N_detected_ was higher than the other outcome measures in both comparisons. The high variance in N_detected_ can be explained by various factors causing the sensitivity differences for detecting arteries across sites. Differences in the reconstruction software of the scanners could likely partially explain this variation. The variation of N_detected_ between sites of the same vendor might reflect variation in patient handling by the local MRI technician, such as the usage of padding to limit motion, which was not standardized and may have varied between sites. Also, despite slice planning was standardized via an illustrated instruction, variations between technicians in the execution of this planning cannot be prevented and could effect the number of visible vessels. Another source of variation could be that the protocol could slightly vary between sites as gradient performance or the availability of (commercial) options in the software could vary and the protocol at each site was implemented as close to the requested parameters as possible. All these factors could have contributed to the variance of N_detected_ and these differences in sensitivity illustrate the challenges in harmonizing the 2D PC-MRI sequence across multiple sites and vendors, as shown in the differences in scan parameters between sites and vendors (Table 3) and SNR_v_ differences (Table 4).

In an early attempt by the EUFIND group, the 2D PC-MRI sequence was also attempted to harmonize for the perforating arteries in the semioval center. However, large sensitivity differences between vendors made the implementation of this sequence in these small (≤ 300µm) perforating arteries difficult (Düzel et al., 2019). These sensitivity differences might also affect the CVs of the outcome measures of the perforating arteries in the basal ganglia. The v_mean_ and velocity PI over the entire dataset were slightly higher than reported for control groups in other studies (Arts et al., 2021b; Brink et al., 2023; Perosa et al., 2022). This can likely be attributed to the high variance in N_detected_ due to the aforementioned sensitivity differences across sites. Smaller arteries with a blood flow velocity just above the noise threshold might not be detected in several sites, which could lead to an overestimation of v_mean_ and velocity PI in these participants. A similar effect has been observed when measuring these perforating arteries at 3T MRI (Arts et al., 2021b).

The mean DSC for the manually drawn ROIs on all scans across all sites and vendors (mean: 0.91; range: 0.69–0.95) was comparable to earlier results on 3T 2D PC-MRI data which was single-vendor only (mean: 0.90; range: 0.85–0.95) (Tuijl et al., 2022). The option to manually censor the results of the automatic artery detection in the basal ganglia was added after the observation that the automatic detection could not always successfully remove in-plane arteries (i.e. arteries not perpendicular to the imaging plane). The lenticulostriate arteries in the basal ganglia are quite tortuous (Ma et al., 2019), which can be a challenge for the automatic in-plane artery removal and deduplication steps in SELMA. The arteries in the semioval center are smaller than in the basal ganglia and are censored better with the automatic censoring steps. The operator can freely choose which arteries to include or exclude with manual censoring. The ICCs after manual artery censoring exceeded 0.75 for all outcome measurements over the entire dataset, indicating that despite the differences in acquisition parameters in the entire dataset, manual censoring yields good to excellent inter-rater reliability. The overall ICC for the velocity PI was higher than the reported ICCs for test–retest reliability by (Schnerr et al. 2017)., suggesting that the SELMA results are probably less dependent on the operator (manual ROI delineation and vessel censoring) than on the measurement noise (scan-to-scan variability).

We found no relation between age or gender and the outcome measures when corrected for sites and vendors using a linear mixed model. In literature, velocity PI of the lenticulostriate arteries has been found to be positively associated with age (Perosa et al., 2022; Schnerr et al., 2017; Vikner et al., 2020), and males had a higher velocity PI in the internal carotid arteries (Tuijl et al., 2020) but not in the lenticulostriate arteries (Perosa et al., 2022) compared to females. The lack of an age effect on the velocity PI in our dataset could be explained by both the differences in acquisition parameters and sensitivity between sites and vendors and the relatively small age range of the study participants in our study (mean age: 59; range: 50–70; standard deviation: 6 years) when compared to the other studies in the literature (Perosa et al., 2022; Schnerr et al., 2017; Vikner et al., 2020). In an additional analysis, we found velocity PI to be associated with N_detected_ in our dataset (Supplementary Table 1). As a result of the differences in acquisition between sites, velocity PI seems to be dependent on N_detected_, which could also explain the lack of an age effect on the velocity PI. The association between N_detected_ and PI was negative, which suggests that a lower N_detected_ means that only the relatively larger vessels (with accordingly higher PI) are detected.

Analysis of cerebral perforating arteries of the basal ganglia and semioval center on 2D PC-MRI data has been performed with several other methods using similar algorithmic steps that have been incorporated in SELMA. These methods are tailored towards the analysis of one single artery at a time, require multiple voxels across the diameter of an artery, or require the need for manual delineation of the perforating arteries (Chengyue et al., 2022; Kerkhof et al., 2023b; Zong & Lin, 2019). One method exists that uses convolutional neural networks to automatically segment perforating arteries (Moore et al., 2022). SELMA has been developed specifically for the automatic analysis of small arteries with subvoxel size and pooling those to obtain metrics from them. Blood flow velocity in the smaller distal branches of the lenticulostrate arteries included in SELMA is more attenuated, which could explain the lower observed velocities (4.0–4.7 cm/s versus 7.0 cm/s (Kerkhof et al., 2023b) and 11.8 cm/s (Chengyue et al., 2022)) and PI (0.27–0.49 versus 0.79 (Kerkhof et al., 2023b)) compared to those obtained with methods that (manually) delineated a larger singular branch of the lenticulostriate arteries.

Arterial pulsatility is an important functional measure in neurodegenerative diseases such as cSVDs and can be quantified in a 2D plane, such as done in this study, or with a 4D flow method. The latter approach has been used in the larger intracranial arteries in cSVDs where associations between pulsatility and cSVDs markers were found (Birnefeld et al., 2020; Björnfot et al., 2024). In the perforating cerebral arteries, 4D flow is more difficult to implement due to the small vessel diameters, thus blood flow velocity in these smaller arteries are computed in a 2D plane and pulsatility is derived from the blood flow velocity alone. Especially in cSVDs, we found that the pulsatility of these small vessels are important markers for disease burden (Brink et al., 2023; Brink et al., 2024).

SELMA and its source code were made publically available and can be installed from: https://github.com/TNI-UMCU/SELMA/ and is provided with step-by-step installation instructions, including a user manual. The tool is supported on Windows and Linux.

This study does have some limitations that have to be addressed. First, the results of our multi-vendor comparison and inter-rater reliability analyses are potentially affected by the differences in hardware, acquisition parameters and reconstruction settings across all sites and vendors. It is unclear how much this could have affected the manual ROI delineation in the basal ganglia or the manual artery censoring. However, these differences are assumed to affect the inter-site and inter-vendor differences in the outcome measures more than the inter-rater reliability. Future work on harmonization of the 2D PC-MRI sequence across multiple vendors, by e.g. using a vendor neutral MR pulse sequence program (Karakuzu et al., 2022; Tong et al., 2022), should address these differences to further stimulate the usage of these measurements of hemodynamic function in the perforating arteries in multicenter studies. Second, the SELMA approach to include subvoxel arteries in size might lead to underestimating the blood flow velocity due to partial volume effects (Bouvy et al., 2016). It is assumed that the effect of underestimation is comparable between subjects and patients, thus still allowing the study of disease or physiology. Third, the relatively low number of included participants per site could also have affected the inter-site and inter-vendor comparisons for all outcome measures. We assume, however, that the contribution of the study population and sample size was small compared to the contribution of the sensitivity differences with regard to the variability in the outcome measures we found.

## Conclusion

We present SELMA, a novel small cerebral artery blood flow velocity analysis tool of 2D PC-MRI data. We achieved good inter-rater reliability of the analysis on data acquired from MRI systems with different hardware, vendor, and scan parameters. Consistent and user-friendly analysis of small cerebral arteries is possible using SELMA. Differences in the implementation of 2D PC-MRI across vendors currently hampers multicenter studies on the hemodynamic function of the perforating arteries in the brain. Still, the tool can be used for single-center studies regardless of vendor or site in which 2D PC-MRI is performed in both patients and controls with the same system and protocol.

## Supplementary Information

Below is the link to the electronic supplementary material.

Supplementary file1 (DOCX 195 KB)

Supplementary file2 (DOCX 14 KB)

Supplementary file3 (PNG 311 KB)

## Data Availability

The data that support the findings of this study are available, upon reasonable request, from the corresponding author.
