# Supplementary material for: Blood Flow Velocity Analysis in Cerebral Perforating Arteries on 7T 2D Phase Contrast MRI with an Open-Source Software Tool (SELMA)
Source: Neuroinformatics. 2025 Jan 22;23(2):11. doi: 10.1007/s12021-024-09703-4 (PMC11754306; doi:10.1007/s12021-024-09703-4)
Supplement: Supplementary file 1 — Supplementary file1 (DOCX 195 KB) [file 12021_2024_9703_MOESM1_ESM.docx]

**Supplementary Material**

Table 1: Results of the groupwise comparison between SELMA and the originally published results^23^.

|  | Original | SELMA |
| --- | --- | --- |
| N_detected_ | 24 ± 6 | 28 ± 6 |
| v_mean_ | 3.9 ± 0.6 | 4.0 ± 0.5 |
| PI | 0.28 ± 0.08 | 0.27 ± 0.07 |

Values are given as mean ± SD. N_detected_ = amount of detected arteries; v_mean_ = mean blood flow velocity of the perforating arteries given in cm/s; PI = pulsatility index

Table 2: Results of the linear mixed model between velocity pulsatility index and N_detected_, corrected for scanner vendor.

|  | Velocity PI​ | |
| --- | --- | --- |
| ​*n = 60* | β [95% CI]​ | p-value​ |
| N_detected_ | -0.013 [-0.026 – -0.0013]​ | 0.04 |

. N_detected_ = amount of detected arteries; PI = pulsatility index


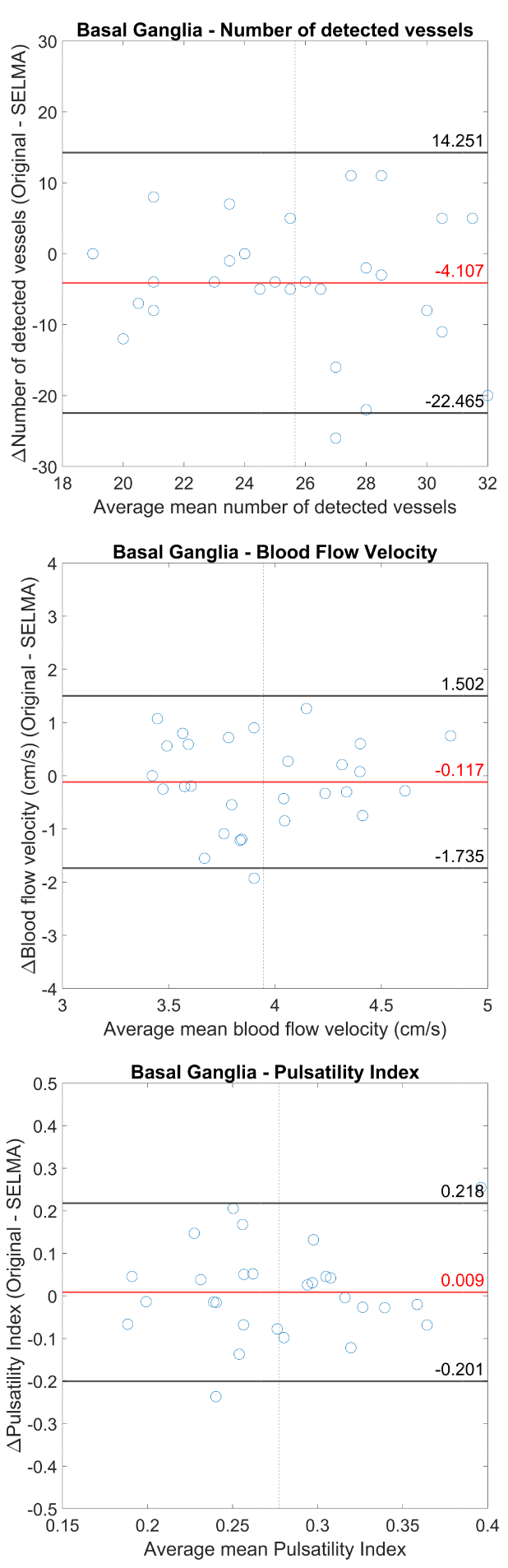


Figure 1 Bland–Altman plots for comparison between the original measurements and SELMA for all outcome measurements in the basal ganglia on previously published data^24^. The red line shows the mean in every figure and the black lines show the limits of agreement.
