# Supplementary material for: Blood Flow Velocity Analysis in Cerebral Perforating Arteries on 7T 2D Phase Contrast MRI with an Open-Source Software Tool (SELMA)
Source: Neuroinformatics. 2025 Jan 22;23(2):11. doi: 10.1007/s12021-024-09703-4 (PMC11754306; doi:10.1007/s12021-024-09703-4)
Supplement: Supplementary file 2 — Supplementary file2 (DOCX 14 KB) [file 12021_2024_9703_MOESM2_ESM.docx]

Table 3: Results of the groupwise comparison between SELMA and the originally published results.

|  | Original | SELMA |
| --- | --- | --- |
| N_detected_ | 24 ± 6 | 28 ± 6 |
| v_mean_ | 3.9 ± 0.6 | 4.0 ± 0.5 |
| PI | 0.28 ± 0.08 | 0.27 ± 0.07 |

Values are given as mean ± SD. N_detected_ = amount of detected arteries; v_mean_ = mean blood flow velocity of the perforating arteries given in cm/s; PI = pulsatility index
