# Supplementary figures and images for: Blood Flow Velocity Analysis in Cerebral Perforating Arteries on 7T 2D Phase Contrast MRI with an Open-Source Software Tool (SELMA)
Source: Neuroinformatics. 2025 Jan 22;23(2):11. doi: 10.1007/s12021-024-09703-4 (PMC11754306; doi:10.1007/s12021-024-09703-4)

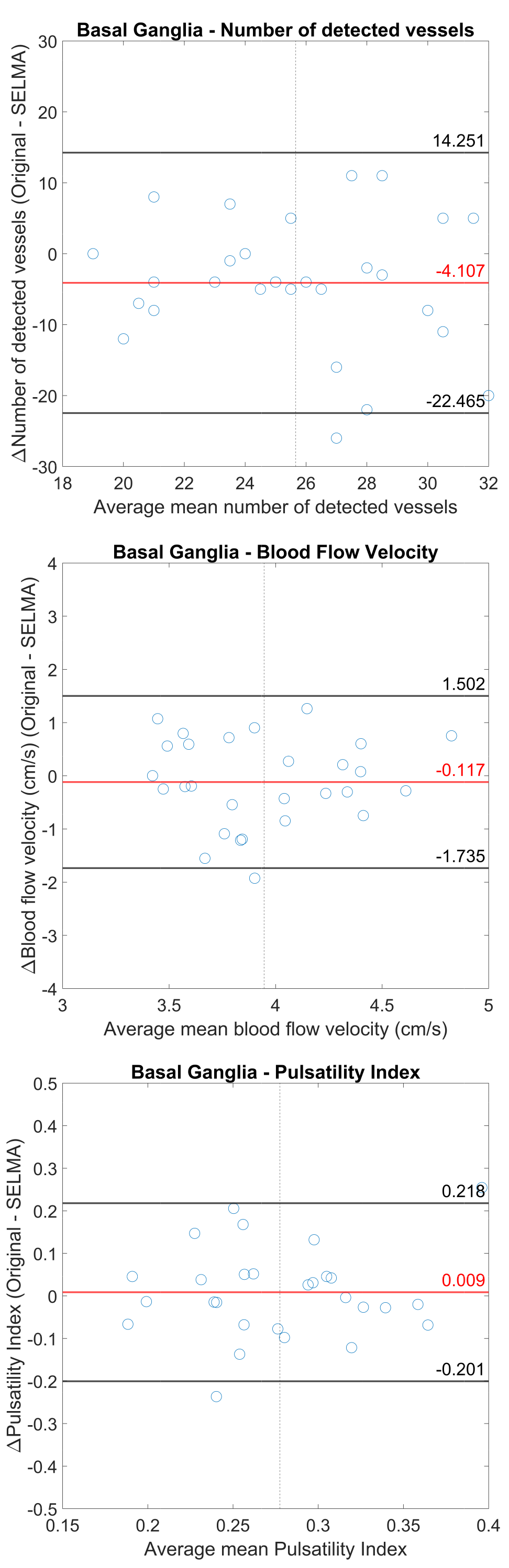

Supplement: Supplementary file 3 — Supplementary file3 (PNG 311 KB) [file 12021_2024_9703_MOESM3_ESM.png]
